# Improving adjustment to daylight saving time transitions with light

**DOI:** 10.1038/s41598-024-65705-x

**Published:** 2024-07-01

**Authors:** Mengzhu Xu, Charikleia Papatsimpa, Luc Schlangen, Jean-Paul Linnartz

**Affiliations:** 1 Lighting and IoT lab, department of Electrical Engineering, Eindhoven University of Technology, Eindhoven, The Netherlands; 2 Signify, Eindhoven, The Netherlands; 3 Human-Technology Interaction Group, department of Industrial Engineering and Innovation Sciences, Eindhoven University of Technology, Eindhoven, The Netherlands

**Keywords:** Circadian rhythms and sleep, Biomedical engineering

## Abstract

Daylight saving time (DST) is currently utilized in many countries with the rationale that it enhances the alignment between daylight hours and activity peaks in the population. The act of transitioning into and out of DST introduces disruptions to the circadian rhythm, thereby impacting sleep and overall health. Despite the substantial number of individuals affected, the consequences of this circadian disruption have often been overlooked. Here, we employ a mathematical model of the human circadian pacemaker to elucidate how the biological clock interacts with daytime and evening exposures to both natural and electrical light. This interaction plays a crucial role in determining the adaptation to the 1 hour time zone shift imposed by the transition to or from DST. In global discussions about DST, there is a prevailing assumption that individuals easily adjust to DST transitions despite a few studies indicating that the human circadian system requires several days to fully adjust to a DST transition. Our study highlights that evening light exposure changes can be the main driving force for re-entrainment, with chronobiological models predicting that people with longer intrinsic period (i.e. later chronotype) entrain more slowly to transitions to or from DST as compared to people with a shorter intrinsic period (earlier chronotype). Moreover, the model forecasts large inter-individual differences in the adaptation speed, in particular during the spring transition. The predictions derived from our model offer circadian biology-based recommendations for light exposure strategies that facilitate a more rapid adaptation to DST-related transitions or travel across a single time zone. As such, our study contributes valuable insights to the ongoing discourse on DST and its implications for human circadian rhythms.

## Introduction

Daylight saving time (DST) is the practice of moving the clock forward one hour from standard time in spring (jump forward), and changing it back again in the fall (fall back). The overarching concept behind this practice is to enable everyone to optimize their utilization of natural daylight, consequently conserving energy by diminishing the requirement for electric lighting during the evening. However, light is also a primary environmental cue (zeitgeber) to synchronize and entrain the central biological clock in the brain that structures and coordinates the 24-h (circadian) rhythms in human physiology, behaviour and sleep-wake patterns. DST was originally designed and promoted for individuals with outdoor lifestyles by increasing their daylight exposure. In contemporary society, human spend most of their time indoors (on average 87%^1^), where they may experience limited exposure to natural light and are particularly susceptible to the effects of DST transitions, which can disrupt circadian rhythms and impair sleep duration and quality^2–5^. These effects may persist for several days after the shift, or even longer^6^. A DST transition abruptly induces a 1 hour discrepancy between the external clock and the central circadian clock within the brain. Although the central circadian clock usually requires several days to fully adapt to this 1 hour shift in clock time upon a DST transition, this was initially regarded as of little consequence. However, a growing body of evidence suggests otherwise.

**Figure 1:**
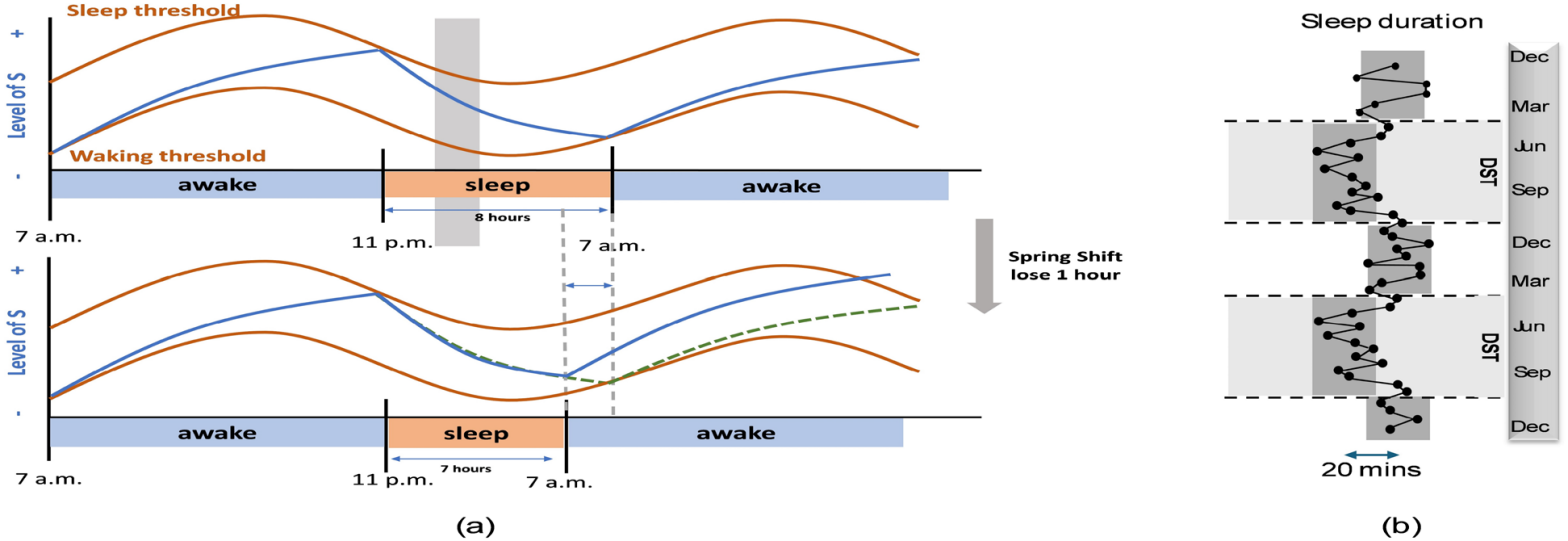
(**a**) The impact of introducing a DST spring shift on people’s sleep based on the two-process model^7^. The blue curve represents the homeostatic process S, which accumulates when people are awake and dissipates during sleep. The orange curve illustrates process C. During the increase of process S, the individual is awake and the sleep pressure builds up. When process S reaches the sleep threshold, the individual falls asleep and decreases until it reaches the wake threshold of process C, which triggers awakening events^8^. The gray square represents the hour “stolen” by the spring shift. The blue line in the bottom figure gives the homeostatic process for a person with fixed bedtimes, which we study persons with self selected bedtimes here (green dashed line). (**b**) Seasonal variations in sleep duration result in approximately 20 min more sleep in winter than in summer (p < 0.0001)^9^: The black dots represent the average sleep duration at biweekly intervals. *DST* is the daylight saving time period.

The switch to DST in spring causes individuals who adhere to strict daily routines to awaken one hour earlier with respect to solar time. This leads to a noteworthy reduction in light exposure during the initial morning hours, while extending natural light exposure in the evening. This change in light patterns is recognized for causing a shift towards later and more varied sleep timings among individuals^10^. Figure 1a illustrates a physiological explanation of sleep curtailment in humans after a spring shift considering the two-process model^7^. The sudden change in light exposure during the DST spring transition closely mirrors the shift in the light-dark cycle experienced when moving from the westernmost to the easternmost part of a time zone. Living on the sunset (western) side of the time zone delays sleep timing and induces a later chronotype in people as compared to living on the sunrise side^11^ and this is associated with reduced health^12^ and an increased difference in sleep timing between work and work-free days (this difference is also denoted as social jet-lag^13^). Moreover, living on the western side of a time zone shortens the duration of sleep by 19 minutes and compromises health and (economic) productivity compared to living on the sunrise side^2,14^. Thomas Kantermann^9^ investigated the seasonal adaptation of the circadian rhythm during DST transitions using a sample of 55,000 subjects from Central Europe. The study revealed a notable alteration in self-reported sleep duration of approximately 20 minutes across DST transitions, as illustrated in Fig. 1b. Zerbini and Merrow^15^ also observed earlier and shorter sleep in summer, a phenomenon attributed to both DST and changes in the photoperiod. These findings collectively suggest that both temporary (during DST transitions) and permanent (perennial) adoption of DST are not advisable. Moreover, on the long term recurring transitions to and from DST maybe expected to have negative implications for human health and well-being^16,17^.

While Pfaff and Weber^18^ report a statistically significant increase in accident frequency and emergency hospital cases following the introduction of DST in the spring in Germany, Lahti^19^ found no significant change in the frequencies of admission rates for accidents and maniac episodes based on nationwide data from the Finnish Hospital Discharge Register. Regarding the increased incidence of traffic accidents, David and Thomas^20^ report a significant increase in traffic injuries in spring for up to two weeks following the clock change but no change in the frequency of accidents after the autumn transition.

Transitions to and from DST have also been suggested to lead to an increased incidence of acute myocardial infarction (AMI). Čulić et al.^21^ report that the incidence ratio for AMI for the first four workdays after the spring DST transition was 1.29 (95% CI 1.09–1.49) and 1.44 (95% CI 1.19–1.69) for the DST transition in autumn. Janszky^22^ report similar findings for the spring transition with an elevated incidence rate of AMI of 1.039 (95% CI 1.003–1.075), while they found no statistically significant change in incidence of AMI after the autumn shift. These studies support the possibility that DST transitions represent a chronobiological challenge that might increase the risk of AMI across the population.

Several studies link sleep disruption following DST transitions with behavioral changes. Gaski and Sagarin^23^ found a surprisingly strong negative relationship between the imposition of the time policy in a geographic area and the scholastic assessment test (SAT) scores of local high school students. Medina et al.^24^ reported that the spring DST onset adversely affects sleep and vigilance in high school students and results in increased daytime sleepiness, longer reaction times and increased lapses.

In summary, there is strong evidence that the 1 hour clock shift resulting from transitions to or from DST induces an extra (temporary) desynchronization between (social) clock time, solar time, and the internal biological clock that has repercussions for human health, functioning, and well being^9,12,21–24^. The goal of this work is to first verify whether a mathematical model for circadian rhythms and sleep behavior produces the same insights that experimental studies reported on the adaptation to DST transitions. Second, we explore other insights, beyond those already verified experimentally, that can be investigated by running the model for a large population of virtual (digital) participants. This approach has potential in finding candidate measures to mitigate the effects DST, before performing a large experimental test campaign. It can also, as we show in this paper, forecast statistical distributions if measures are productive or counterproductive for individuals with specific intrinsic parameters. We further believe that a verification of whether the model has adequate predictive value may pave the way to personalized light exposure plans.

## Results

### Increasing daytime illuminance enhances the adjustment to DST transitions

**Figure 2:**
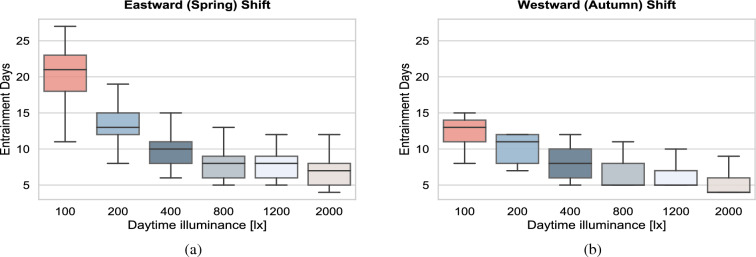
Time required to fully adjust to the new time (zone) following a DST transition for a cohort of 200 simulated individuals exhibiting intrinsic circadian periods distributed normally, with mean values (±SD) of $24.2 \pm 0.2$ h^25^. Illuminance refer to corneal light exposure (i.e. at the eye) for daytime (wake-19:00) illuminance ($L_1$) set to the values indicated on the x-axis (distinguished with color). Evening light exposure $L_2$ was simulated at 35 lx from 19:00 to sleep. (**a**) Results for spring (traveling eastward) DST transition. (**b**) Results for autumn (traveling westward) DST transition. The central (black) line indicates the median, and the bottom and top edges indicate the 25*th* and 75*th* percentiles, respectively.

First, we considered a light-dark cycle with evening light ($L_2$) set at 35 lx and a constant daytime illuminance ($L_1$) that was varied between 0 to 2000 lx and calculated the distribution of the time required to fully adjust to the new time zone for different illuminance (Fig. 2a for the spring and Fig. 2b for the autumn DST transition respectively). In this context, “daytime” denotes the time period between the time that the model wakes up and up to 19:00, while “evening” refers to the time period between 19:00 up to the time that the model falls asleep. To mimic the self-selection of light under realistic conditions, the duration of the photoperiod in the simulations is solely determined by people’s sleep-wake behavior. Therefore, changes in the natural photoperiod between summer and winter are considered to have a negligible impact; people with different circadian intrinsic period will produce different light profiles, due to a different timing and amount of wakefulness (i.e. time spent awake). The model predicts that when individuals are exposed to bright daytime illuminance, they adjust more readily to the DST transition. Next to that, the inter-individual variability in time needed to adjust is lower when the daytime illuminance is high. Under bright daytime light, for example, 800 lx at eye level, the time required to fully adjust to the DST spring transition (Fig. 2a) is distributed with means (±SD) of 7.9 ± 2.4 d. This distribution is considerably shifted as the light levels decrease. For example, under typical office illumination (~200 lx on average, measured vertically in the eye position), the time required to adjust to the spring DST transition is distributed with means (±SD) of $13.5 \pm 4.4$ d. These model predictions are in good agreement with experimental studies that found large inter-individual differences in DST spring-change adjustment ranging from a few days up to 2 weeks^9^.

### Adjustment to the DST transition in autumn is more rapid than in spring

Most individuals adapt more readily to delays of the clock than to advances, for instance people usually suffer less from jet lag after westward than after eastward flights^26^. A similar pattern is predicted by our simulation for the DST transitions as depicted in Fig. 2b. Our results show that the adaptation process is faster for the autumn DST transition. For the autumn transition, the corresponding transition time under 200 lx is distributed with means (±SD) of $10.1 \pm 2.1$ d (compared to $13.5 \pm 4.4$ d that are required for the spring transition). These model predictions corroborate experimental studies^4^ that reported on the differential effect of the spring and autumn transitions with a stronger deterioration of the sleep/wake cycle quality after the spring as compared to the autumn DST transition. The model also predicts that a higher daytime illuminance not only reduces the time required to adapt to the (DST induced) 1 hour time shift, but also reduces the inter-individual differences in establishing full adjustment.

### Reducing evening light exposure helps adjusting to the spring DST transition

**Figure 3:**
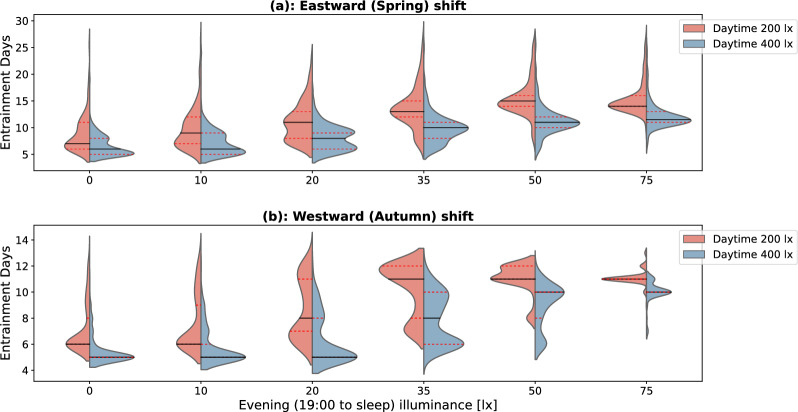
Violin plots showing the time required to fully adjust to the DST transitions for a population of 200 simulated individuals. The illuminances refer to the corneal light exposure for evening (19:00 till sleep) illuminance ($L_2$) set to the values indicated on the x-axis. Two different daylight settings ($L_1$) are considered (200 lx and 400 lx) (**a**) Results for spring (traveling eastward) DST transition. (**b**) Results for autumn (traveling westward) DST transition. The black line represents the median and the red dotted line shows the 25*th* and 75*th* percentiles. The y-axis scales are not uniform between the two subplots.

Here, we investigate a spring transition under two different daytime (wake-19:00) illuminance ($L_1$= 200 and 400 lx respectively), and for each daytime illuminance, the evening (19:00-sleep) illuminances $L_2$ was varied between 0 and 75 lx. In our observation, when there is insufficient difference between daytime and evening light, the model may fail to entrain. This phenomenon has also been recognized and discussed in previous research^27^. Results are presented only for combinations of daytime-evening illuminance and intrinsic circadian periods for which the model entrains to 24-hour rhythms. The model predicts that a higher daytime illuminance not only shortens the time needed to fully adjust to the spring DST transition but also reduces the inter-individual differences, as illustrated in the top violin plot in Fig. 3.

The speed of adaptation to the DST spring transition is significantly reduced when exposed to a higher evening illuminance (Fig. 3). Under typical daytime illuminance (~400 lx) and low evening illuminance, say 10 lx, 50% of the adjustment to the 1 hour time shift is achieved within the 5 d following the DST transition, and after 7 d, 75% of the population achieves full adjustment or re-entrainment. The adjustment speed significantly drops when individuals are receiving higher light levels in the evening. As an illustration, assuming each individual’s eye is exposed to 50 lx of light during the evening, it would require approximately 10 d for 50 percent of the population to attain complete adaptation to the 1 hour shift induced by spring DST transition. Under daytime illuminances that are quite common for many workplace settings indoors (~ 200 lx) and modest evening illuminance, say 10 lx, the time required to adjust to the spring DST transition shows a diffuse distribution with mean (±SD) of $9.8 \pm 3.9$ d. This is decreased when exposed to higher evening light levels; for instance, when receiving 50 lx at the eye, the model adjusts with a mean (±SD) of $15.4 \pm 3.1$ d (p-value < 0.0001 on Mann-Whitney *U* test).

Similar patterns can be found for the autumn DST shift. During autumn transitions, a comparable trend arises, wherein the period necessary for adaptation slightly elongates with the increased intensity of evening exposures. Under 200 lx daylight setting, individuals having a 10 lx evening light take $7.6 \pm 2.2$ d to fully adjust, while those exposed to 50 lx during the evening require $11.0 \pm 1.2$ d for full adaptation.

Our model simulation results in Figs. 2 and 3 can be explained from the fact that in our simulations the daytime illuminance $L_1$ starts at the self-selected wake up time of the model and continues to 19:00 in the evening where the illuminance drops to a lower value ($L_2$) which is maintained until the self-selected bedtime. Due to starting $L_1$ at the self-selected wake time, the light exposure in the phase advancing morning portion of the phase response curve (PRC)^28^ does not really change upon a DST transition (see also Fig. 7). This implies that the $L_1$ to $L_2$ drop at 19:00 in the evening is the main driving force for phase shifting (and re-entrainment) upon a DST transition. During the spring DST transition, the clock time of the minimum core body temperature CBT (CBT_min) abruptly increases by 1 hour, so that the $L_1$ to $L_2$ drop at 19:00 in the evening suddenly occurs 1 hour further away from the CBT_min. This renders the (evening) light exposure profile less phase delaying (see Ref.^28^) as compared to before the spring DST transition (i.e. the spring DST transition induces a net phase advance). During the autumn DST transition, the clock time of CBT_min abruptly drops by 1 hour, so that the $L_1$ to $L_2$ drop at 19:00 suddenly occurs 1 hour more close to the CBT_min. This renders the (evening) light exposure profile more phase delaying as compared to before the DST transition (i.e. the autumn DST transition induces a net phase delay). For both DST transitions a larger $L_1$ to $L_2$ drop results in a larger driving force for re-entrainment. This can be seen in Figs. 2 and 3: higher daytime illuminances $L_1$ and/or lower evening illuminances $L_2$ result in faster re-entrainment for both DST transitions.

**Figure 4:**
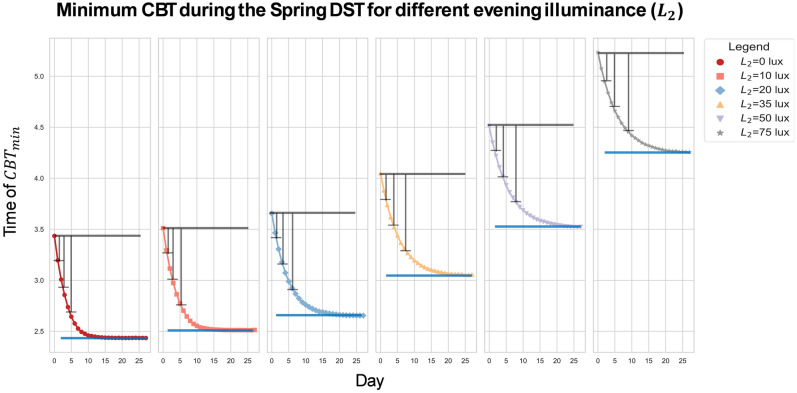
Circadian phase, represented by the time of minimum core body temperature, across the days following the spring DST transition of 200 individuals with average intrinsic period ($\tau$=24.2). Results are presented for daytime (wake-19:00) light illuminances ($L_1$) of 400 lx, and evening (19:00-sleep onset) illuminance ($L_2$) set to the value corresponding to the symbol (colour) shown in the legend. The black horizontal line indicates the time of the minimum in core body temperature (CBT) immediately after the DST transition. The blue horizontal line marks the time of the minimum in CBT immediately before the DST transition (baseline), it also marks the time of the CBT min after full re-entrainment of the model to the DST transition. The consecutive dots in each sub-plot indicate the time of (CBT_min) corresponding to each day after a DST transition. Whiskers indicate on which days 25%, 50% and 75% percentage of the 1h shift needed to accomplish full re-entrainment is covered.

Figure 4 depicts the process by which individuals undergo adaptation from a CBT_min perspective. Under typical light setting (daytime light $L_1$=400 lx and evening light $L_2$=10 lx), 25% of the adjustment to the 1 hour time change occurs within the first day following the spring DST transition, with 75% of full re-entrainment achieved after 5 d. However, the speed of adaptation notably diminishes with increased evening light exposure. For example, when individuals are exposed to 75 lx at the eye during the night, it takes 2 d to achieve 25% of the 1 hour shift and 9 d to reach 75% adaptation.

### A morning walk helps adjusting to the spring DST transition

Here, we simulate a light profile with a fixed daytime (wake-19:00) illuminance $L_1$ = 400 lx and an evening (19:00-sleep) illuminance $L_2$ of 35 lx. In addition, on the four days succeeding the DST transition, we included a 30-minute bright (outdoor-like) light exposure under $L_3$ = 10000 lx that started 1 hour after wake-up of the model.

**Figure 5:**
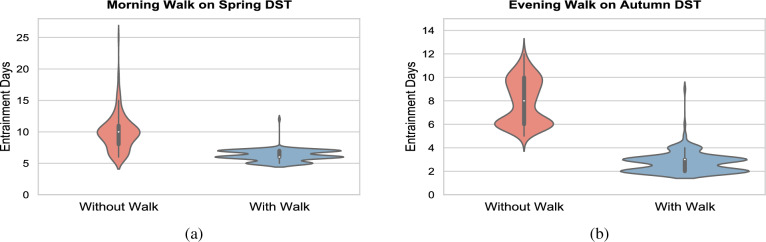
Violin plot representing the effect of a(n) morning/evening walk on the time required to fully adjust to the new time zone following a spring/autumn DST transition for a cohort of 200 simulated individuals exhibiting intrinsic circadian periods distributed normally, with mean values (±SD) of $24.2 \pm 0.2$ h^25^. Results are presented for daytime (wake-19:00) illuminance ($L_1$) of 400 lx, and evening (19:00-sleep onset) illuminance ($L_2$) of 35 lx. These illuminances refer to corneal light exposure, specifically at the eye. Each violin plot shows the distribution of the values as well as the median as a white dot, the interquartile range as a thick black bar, and the 95% confidence interval as a thin black bar.

Our results suggest that even only half an hour of exposure under bright light (e.g. a morning walk outdoors on a sunny day) can significantly reduce the time required to fully adjust to the DST transition and can also reduces inter-individual differences in re-entrainment. Under a typical daytime (indoor) illuminance ($\sim 400 \, \text {lx}$) and a $35 \, \text {lx}$ evening illuminance, the time required to adjust to the spring DST transition shows a wide distribution with a mean (±SD) of $10.1 \pm 3.5$ d, as shown in Fig. 5a. The simulations indicate that having a morning walk significantly reduces the time needed to adjust to the DST transition and its inter-individual variability, towards a mean (±SD) of $6.5 \pm 2.8$ d.

### An evening walk speeds-up adjustment to the autumn DST transition

We again consider a light profile with a fixed daytime (wake-19:00) illuminance $L_1 = 400$ lx and an evening (19:00-sleep) illuminance $L_2$ of 35 lx. In addition, we included a 30-minute outdoor-like light exposure under $L_3 = 2000$ lx starting at 19:00 after the autumn DST transition. Our results suggest that even only half an hour of exposure under bright light (e.g. a walk outdoors after work) can significantly reduce the time required to fully adjust to the DST transition and the inter-individual variability, see Fig. 5b.

Under a typical daytime (indoor) illuminance ($\sim 400$ lx) and a 35 lx evening illuminance, the time required to adjust to the autumn DST transition shows a distribution with a mean (±SD) of $7.9 \pm 1.9$ d. The simulations indicate that having an evening walk effectively reduces the time needed to adjust to the autumn DST transition , towards a mean (±SD) of $2.7 \pm 0.9$ d.

### Wearing sunglasses in the morning speeds-up adjustment to the autumn DST transition

Here, we consider a light profile with a fixed daytime (wake-19:00) illuminance $L_1$ = 400 lx and an evening (19:00-sleep) illuminance $L_2$ of 35 lx. In addition, on the four days succeeding the autumn DST transition, we included a 2-hour light exposure under $L_3$ = 80 lx upon waking up (assuming that typical CAT3 sunglasses have a typical transmission of about $20\%$). The model predicts that reducing morning light in the autumn DST transition is as effective as going outdoors in the evening for a walk (but need 2 hours compare to half hour evening walk).

With the same light setting as evening walk experiment ($L_1$=400 lx and $L_2$=35 lx), the time required to adjust to the autumn DST transition follows a distribution with mean (±SD) of $7.9 \pm 1.9$ d. The simulations indicate that wearing sunglasses during the first 2 hours after waking up significantly reduces the time needed to adjust to the DST transition and its inter-individual variability, towards a mean (±SD) of $3.5 \pm 0.8$ d.

### The differences in adaptation to DST among people with different chronotypes

**Figure 6:**
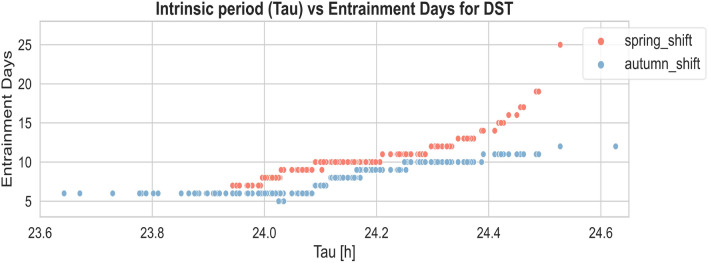
Scatter plot, representing the effect of the intrinsic period $\tau$ on the time required to adjust to the spring/autumn DST transition for a population of 200 simulated individuals with a normally distributed intrinsic circadian period (24.2 ± 0.2 h) in each experiment. Results are presented for daytime (wake-19:00) light illuminance ($L_1$) of 400 lx, and evening (19:00-sleep onset) illuminance ($L_2$) of 35 lx.

One of the most widely accepted hypotheses for explaining the variances in circadian entrainment among chronotypes involves disparities in the intrinsic circadian period^29^. The intrinsic period of endogenous oscillators, while not precisely 24 hours, undergoes entrainment by zeitgebers on a daily basis. Typical intrinsic period lengths are 24.3 hours for individuals with an evening (i.e. late) chronotype and 24.1 hours for those with a morning (i.e. morning) chronotype^30^. Here, we investigate the effect of this intrinsic period ($\tau$) in DST transitions. As depicted in Fig. 6, we employed a light setting with daytime (wake-19:00) light illuminance ($L_1$) of 400 lx, and evening (19:00-sleep onset) illuminance ($L_2$) set to 35 lx. The plots illustrate that there is significant individual variation when facing a DST transition. This suggests that with an increasing intrinsic period, more time may be required to adjust to both DST transitions in spring and autumn.

**Figure 7:**
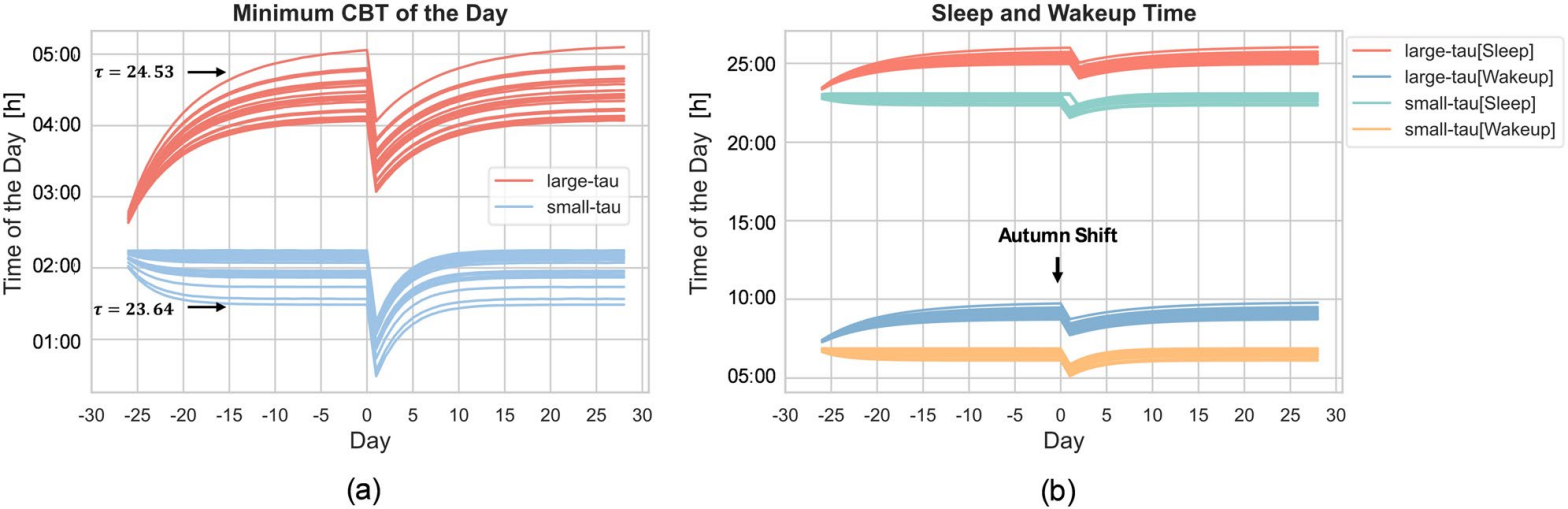
(**a**) The plot illustrates the timing of the minimum CBT for individuals exhibiting extremely small intrinsic periods in blue ($\tau$ ranging from 23.64 to 23.91) and those with extremely large intrinsic periods in red ($\tau$ ranging from 24.36 to 24.53) during an autumn DST shift. The autumn shift happens at day 0, the days before the day 0 is utilized to facilitate the convergence of the personalized day–night rhythm for the mathematical model. (**b**) The plot depicts the natural sleep onset and wake-up times for simulated individuals using the sleep-wake state from the two-process model. The black dotted line indicates the alarm set for 7 a.m.

However it is somewhat counter-intuitive that later chronotype/evening persons (with their longer intrinsic period) need more time to adjust to autumn DST transition as compared to earlier chronotypes/morning persons. To understand this, we conducted an investigation into the sleep and wake-up times, as well as the timing of the minimum in core body temperature (CBT_min) of simulated individuals, as depicted in Fig. 7. We selected the most extreme 20 individuals for each of the morning-type and evening-type populations in our simulations for the investigation. It is assumed that evening persons adjust faster to the autumn DST shift since they naturally wake-up late and therefore better fit the 1 hour delay. However, in our simulation we came to a different conclusion, which can be explained as follows. Figure 7 demonstrates that CBT_min and the self-selected wake up and sleep (onset) times in the model closely follow each other and experience a similar shift upon a DST transition. This implies that the phase advancing effect of the morning portion of the light profile does not change upon a DST transition: in our light profile choice of Figs. 2, 3 and 6, the evening light is driving the re-entrainment to the DST transition. As explained earlier, in our light profile choice, the $L_1$ to $L_2$ drop at 19:00 in the evening is the main driver for phase shifting and re-entrainment upon a DST transition: a DST transition results in an abrupt reduction (in spring) or increase (in autumn) of the phase delaying effect of the (evening) light exposure profile as compared to before the transition. People with a shorter tau (earlier chronotypes) have an earlier timing of CBT_min (see Fig. 7a) and for them the $L_1$ to $L_2$ drop at 19:00 occurs more close to CBT_min as compared to people with a longer tau (later chronotypes). This makes that people with a shorter tau experience larger phase shifts and adjust more rapidly after either DST transition as compared to people with a longer tau, as shown in Fig. 6.

The importance of the timing of the $L_1$ to $L_2$ drop in the evening for re-entrainment upon a DST transition is further highlighted by adding an extra hour of $L_1$ light of 400 lx (from 19:00 to 20:00) to the model. The addition of one extra evening hour of $L_1$ moves the $L_1$ to $L_2$ drop more close to CBT_min and therefore shortens the entrainment duration for autumn DST transitions. For the autumn transition, one extra hour of $L_1$ shortens entrainment from 12 to 5 d for an evening person (with $\tau = 24.53$) and from 6 to 3 d for a morning person (with $\tau = 23.64$) .

### Learning realistic light patterns from field study

**Figure 8:**
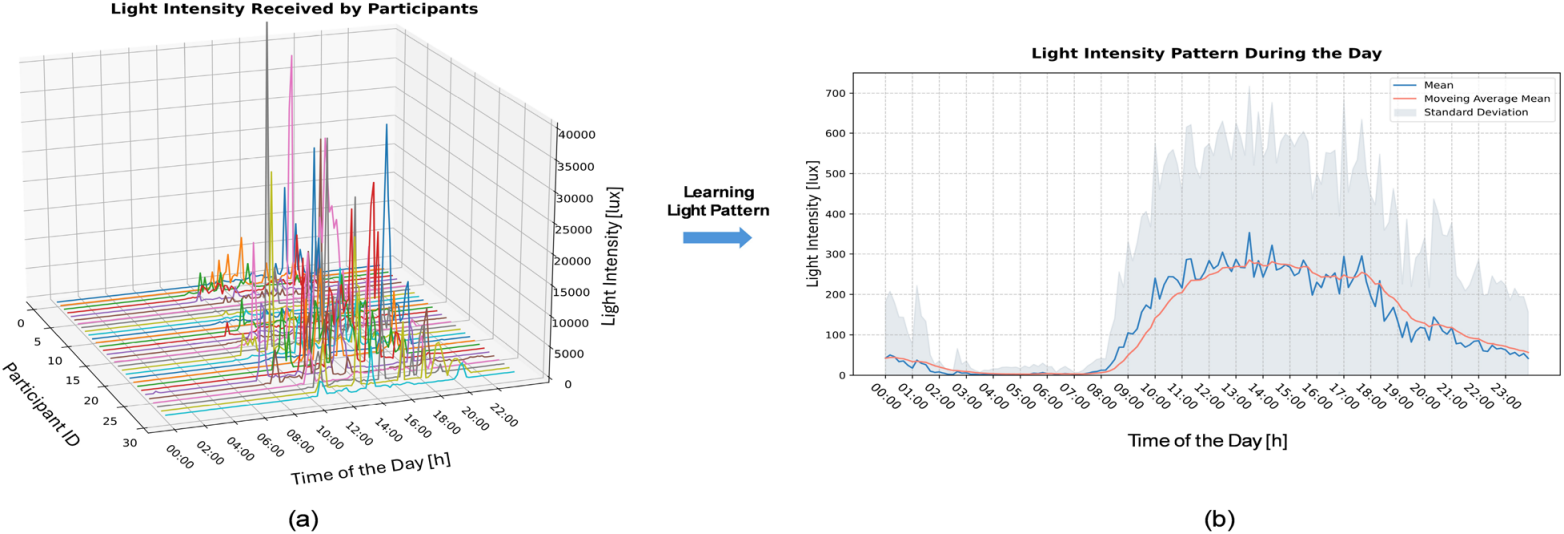
(**a**) depicts the light intensity received by the participants in our field study throughout the day. Noticeable peaks are evident, attributed to outdoor sunlight exposure. (**b**) showcases the observed light patterns derived from the field study data. The blue line signifies the mean light intensity, with outliers filtered out. The light blue shaded area indicates the standard deviation, while the red line represents the smoothed mean value achieved through a moving average method (with parameters: $step=1, weight=0.8$).

In order to further validate the appropriateness of the fixed lighting settings used in our simulation experiments, we conducted an analysis of real-world lighting intensity data obtained from individuals over the course of a day. Figure 8a presents lighting intensity data that we collected at different times of the day from a field study in the Netherlands (This field study, spanning approximately one year from October 2021 to September 2022, engaged 25 participants. For each participant, three consecutive weeks data was measured using MotionWatch 8 devices). Typically, around 8:00, individuals are exposed to the first relatively strong light exposure of the day (most likely sunlight received when leaving home in the morning). Throughout the day, individuals are typically exposed to lighting intensities ranging from 200 to 400 lx indoors, with occasional peaks of high-intensity light resulting from limited outdoor activities. Around 19:00 in the evening, the light intensity gradually decreases to below 100 lx, resembling evening indoor lighting, until bedtime. In our simulations, we set $L_2$ to 35 lx to mimic the light during the evening (also explained in the Methods section). After performing data cleaning and pre-processing, we removed outliers from the data (based on the 5*th* and 95*th* percentiles), followed by calculating the mean and standard deviation for each time point. To obtain a smoother lighting intensity profile, we employed a moving average approach to process the data, as depicted in Fig 8b. The moving average lighting profile derived from the field study data suggests that using a two-stage constant light ($L_1$ for daytime and $L_2$ for evening) to approximate the amount of light a person receives over the course of a day is reasonable, which is the approach designed and employed in the simulations for this study.

**Figure 9:**
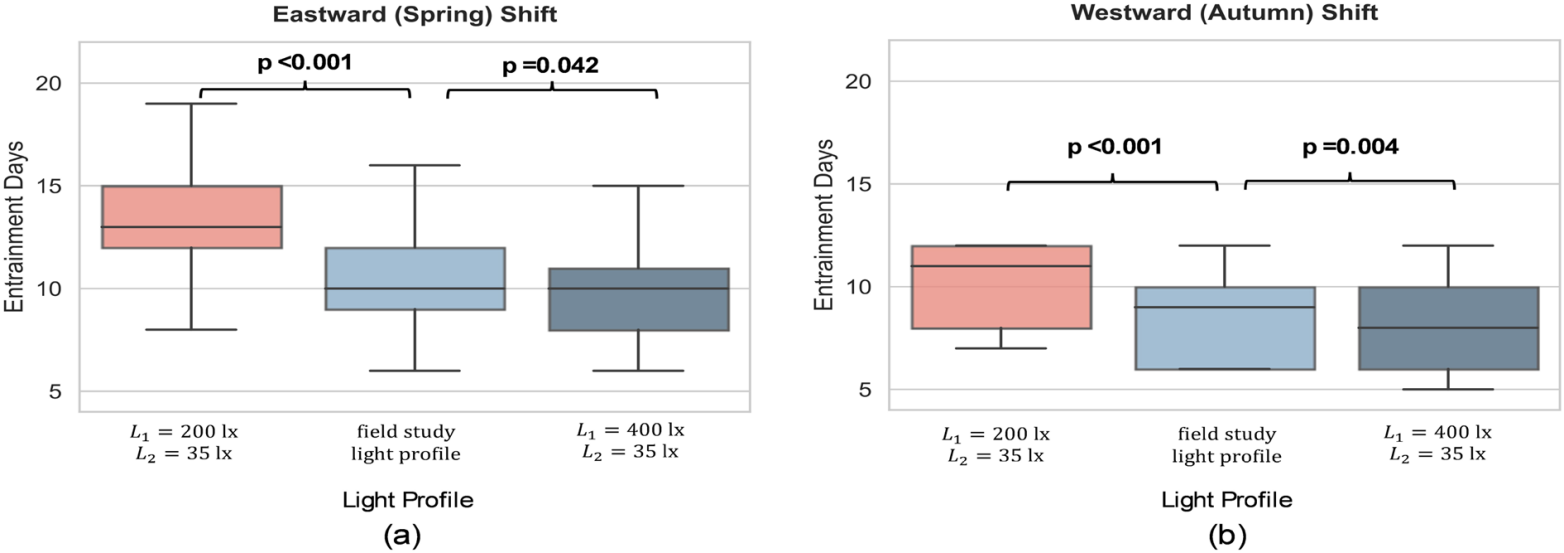
Boxplots show the comparison between different lighting profile settings and the real light pattern derived from field study at different DST transitions. Three different light settings were employed: (**1**): $L_1$=200 lx and $L_2$=35 lx; (**2**): sampled light profile learned from field study; (**3**): $L_1$=400 lx and $L_2$=35 lx). (**a**) Results for spring (eastward) DST transition. (**b**) Results for autumn (westward) DST transition. The Mann-Whitney U test is applied to test the similarity between the different light settings. The results indicate the real light profile performs closer to daylight $L_1=400$ lx scenario.

To further explore this, we sampled the light intensity profiles learned from field study at 30-minute intervals and applied them to experiments for both the spring and autumn DST transitions. Three different light settings were employed: (A): $L_1$=200 lx and $L_2$=35 lx; (B): sampled light profile learned from field study; (C): $L_1$=400 lx and $L_2$=35 lx). These settings were tested on the same population for both DST transitions, and the results are shown in Fig. 9. Our analysis indicates that the population exposed to the real light profile (learned from field study) align more closely with the condition of $L_1=400$ lx and $L_2=35$ lx compared to those exposed to $L_1=200$ lx and $L_2=35$ lx.

## Discussion

Within a time zone, the human circadian system and the endogenous sleep-wake cycle are reported to entrain to solar time^31^. During transitions into or from DST, the light-dark cycle abruptly shifts by 1h, and our results highlight that the adjustment of the internal biological clock in the brain to this abrupt 1h time change is not instantaneous. Several days might be necessary to adapt and re-entrain the internal sleep-wake cycle to the 1h time shift of the external clock, and there can be significant variability among individuals in this process of synchronization. The time needed to adjust merits to be minimized as this will reduce the negative impact of DST transitions on our society and it will also limit disruptions in sleep and behavior during the adaptation to such abrupt 1h shifts in the light-dark cycle.

Ideally, in the first week after the spring DST transition, it is advisable to avoid scheduling classes and school performance tests during the first school hours. Examination performance of teenagers is known to be sub-optimal in the first morning hours, especially for later chronotypes^32^. This effect will be even more pronounced in the week following the spring DST transition. Our study, as illustrated in Fig. 6 suggests that individuals with later chronotypes may require up to an additional two weeks to fully adjust to the spring DST transition, potentially resulting in poorer examination performance.

One may wonder whether the temporary (i.e. during spring/summer only) or per annual adoption of DST is healthy from a chronobiological perspective. During DST transitions, the solar time is effectively delayed by 1h, which can be considered equivalent to moving from the most east side of a time zone to its most west side. Living further west in a time zone is associated with more health problems, a higher risk of cancer, and shorter sleep duration^14,16,17,33^.

More recently, Arguelles-Prieto et al.^34^ applied a human response curve to light during DST transitions, using data from a wearable monitoring device to assess circadian phase. They found that transitioning to DST disrupts circadian markers more than transitioning back. The self-selected light profile observed in their study aligns well with the light profile derived from our field study. Another study^35^ reported that individuals genetically predisposed to a morning tendency adjusted to the spring DST transition in a few days, while genetically predisposed evening-inclined individuals had not shifted within the first week. These experimental results corroborate with our model predictions that transitioning to DST requires more time to adjust and individuals with a longer intrinsic circadian period require more time to adjust to the DST transitions.

In this work, we employed mathematical models to simulate human circadian rhythms and derived lighting profiles that suit our simulations from the field study. This approach allowed us to investigate how individuals with varying circadian periods adapt to both DST transitions by observing CBT_min. We analyzed a population of 200 individuals with normally distributed circadian periods. However, accurately determining the intrinsic period is crucial yet challenging. Imran et al.^36^ employed a phase-only parametric model to characterize the impact of dim light-dark cycles on period assessment, offering potential integration into future DST-related studies to establish personalized lighting strategies for DST transitions. While we employed the widely accepted Jewett-Forger-Kronauer model in our research, it’s worth noting its reliance on parameters and exclusive focus on light effects. Hence, we recommend exploring alternative modeling approaches as well, such as agent-based models or stochastic models, which can capture the complex interactions between different factors influencing circadian rhythms.

Although dim light melatonin onset (DMLO) and CBT_min are considered the gold standards for assessing circadian phase, other important biomarkers, such as cortisol, also merit further research. Hadlow et al.^37^ suggest that cortisol rhythms remain stable despite changes in daylight saving time, likely because cortisol peaks strongly correlate with awakening time, typically occurring 20 to 30 minutes after waking. Mark et al.^38^ found that the morning cortisol awakening response is closely linked to rising time and less so to the DLMO phase. Investigating the behavior of different biomarkers under the same conditions is important to understand their relationships in the future studies.

## Limitations

The current simulations are tailored to individuals with self-selected bedtimes and an indoor lifestyle, assuming a constant indoor illuminance throughout the day, thereby neglecting variations in daylight entry. Notably, findings by Smolders et al.^39^ from a field study among office workers indicate that exposure to bright light (>1000 lx in the eye) average only a few minutes per hour. Consequently, these simulations may not be applicable to scenarios where individuals spend significant time near windows without blinds, or for individuals with fixed bedtimes (using alarm clocks). Additionally, the simulations do not account for changes in the times of dawn and dusk, potentially impacting light exposure around the DST transitions, particularly during the spring transition. Furthermore, our analysis assumes strict adherence to self-selected bedtimes and fixed work-life cycles, such as finishing work before 19:00 and having access to evening lighting. However, this assumption disregards variations that occur during weekends or among children. Hence, this oversight could affect the observed adaptation times in our experimental findings when extrapolated to real-life contexts.

In the simulations, we adopted ode23 for fitting differential equations of the circadian system due to computational limitation. We confront limitations including precision constraints, struggles with stiff systems, and challenges in capturing certain solution characteristics. Additionally, parameter selection is critical, and the method may fall into local optima.

It has been reported that the homeostatic sleep pressure during wake can depend on the light exposure^40^ and also increases at a slower rate in older people compared to younger ones^41^, and the amplitude of circadian rhythm diminishes with age^42^. In the mathematical equations of the two-process model (process C & S), these age-related changes can be represented by adjusting the parameters $\mu$ and $v_{vc}$. Previous research^27^ indicates that for individuals aged 30, $\mu =4.20 \& v_{vc}=3.37$, while for those aged 17, $\mu =4.60 \& v_{vc}=4.00$ best fit in their experiments. However, in our prior study^43^, we identified a different parameter combination, $\mu =4.00 \& v_{vc}=2.90$ as the best fit based on our field study data. Notably, we maintained this parameter setting throughout our experiment, thus not accounting for the influence of age in our analysis, which may introduce some limitations to the study.

A key aspect is that we have employed a mathematical modeling approach in this study. We verified qualitatively that many of our findings agree with earlier reported experimental outcomes, however an exhaustive verification of the model outcomes is not within reach. Nevertheless, a specific verification of some of the new insights is recommended before implementation, e.g. the adopted/recommended light interventions by wearing sunglasses or walking.

Although we verified that our experimental lighting setup was reasonable in terms of both light intensity visualization (see Fig. 8) and DST adaptation speed (see Fig. 9), the modeling of real and dynamic light profile remains an area requiring further exploration for better simulation results.

## Summary and conclusion

Switching into and out of DST induces a temporary misalignment between the endogenous sleep-wake rhythm and the external clock cycle that has repercussions for performance, sleep, health and well-being. Light exposure is the most important environmental stimulus to overcome this misalignment and fully re-entrain the internal circadian rhythm to the 1 hour shift of our biological clocks.

We confirm that a mathematical model of the human circadian pacemaker can harmonize many earlier findings and capture these in a unified tool. Inspired by the qualitative agreement of the model with experiments reported in the literature, we simulated further effects and tested the effect of a number of individual light exposure interventions. We show that there are large interindividual differences in the time needed to adjust the internal sleep-wake cycle to the abrupt 1 hour shift of the light-dark cycle (and CBT_min) that arises from a DST transition. However, when evening light exposure changes are the primary catalyst for re-entrainment (as will be the case for most situations with self selected bedtimes), chronobiological models predict that individuals with a longer intrinsic period (i.e. later chronotype) entrain more slowly to transitions to or from DST as compared to people with a shorter intrinsic period (earlier chronotype). Importantly, these interpersonal differences are largely associated with personal parameters, such as intrinsic period $\tau$, suggesting the potential for personalized therapeutic interventions.

Under our simulation setting, we observed that an increased daytime illuminance can help to adapt more rapidly to the DST transition and reduces inter-individual differences in adaptation. For instance, when increasing the daytime (i.e. from wake to 19:00) illuminance from 200 to 800 lx, the mean time (±SD) needed for full adaptation to the DST transition can decrease from $13.5 \pm 4.4$ to $7.9 \pm 2.4$ d for the spring transition and from $10.1 \pm 2.1$ to $6.6 \pm 1.8$ d for the autumn transition.

We proposed that having a 30-minute bright light exposure 1 hour after waking up (for instance, by means of a morning walk or bright light therapy) can reduce the time needed to adjust to the spring DST transition (and its SD/inter-individual variability) from $10.1 \pm 3.5$ to $6.5 \pm 2.8$ d. Having a 30-minute light exposure in the evening (for example, by means of commuting back from work before sunset) can reduce the time needed to adjust to the autumn DST transition (and its SD/inter-individual variability) from $7.9 \pm 1.9$ to $2.7 \pm 0.9$ d. Wearing a typical CAT3 sunglasses in the morning on the four days succeeding the autumn DST transition can reduce the time to adjust to the transition from $7.9 \pm 1.9$ to $3.5 \pm 0.8$ d. Our model findings corroborate experimental studies^4,9^ and provide a good basis to define optimal light-based strategies that facilitate adjustment to DST transitions and reduce adjustment time, as well as its inter-individual variability across the population.

To facilitate a better and more rapid adaptation to the transitions into and from DST that continue to be adopted in many countries, the following suggestions might be helpful: (1) Increase the daytime light exposure/illuminance (for instance, by going outdoors, being close to a window, or using higher levels of electrical light). (2) To shorten the time needed to adjust to the spring DST transition, have a 30-minute morning walk (or use a bright light therapy device, which can result in a light exposure up to 10000 lx) early in the day, say 1 hour after waking up, and reduce the evening illuminance. (3) To shorten the time needed to adjust to the autumn DST transition: use a walk or a bright light therapy device in the evening (for instance, after work) and/or wear sunglasses in the first hour after waking up. (4) Individuals with an evening chronotype may require additional time to adapt to DST transitions during both spring and summer, particularly if they adhere to a daily schedule with self-selected bedtimes and consequently receive a day-night light pattern similar to our simulation setting (e.g. receiving office daytime light and change to evening light of $L_2$ at 19:00). In such cases, a later start of the evening light $L_2$ or personalized light therapy could be considered to facilitate a more rapid adjustment process. Moreover, a study by Charikleia^44^ shows how such personalized light concepts may even be implemented in the workplace.

## Methods

We employ a mathematical model and validated it by comparing the model prediction with widely cited findings reported in literature. After gaining trust in the predictive capabilities of the model, we explored further effects, beyond those already known from experiments.

### Entrainment simulations

To model the adaptation of the human circadian system to DST transitions, we employ quantitative mathematical models previously utilized and described in our research^45^. Specifically, we utilize an adapted version of the Jewett-Forger-Kronauer model^46^ to simulate circadian entrainment. This model incorporates inputs such as light exposure and intrinsic circadian period ($\tau$) to estimates circadian phase, represented by the time of nadir in the human core body temperature cycle (referred to as CBT_min). Additionally, we utilize a modified Phillips-Chen-Robinson model^47^ proposed by Skeldon et al.^27^ to simulate the sleep regulation by considering the interaction between circadian mechanisms and the homeostatic process under the influence of light. The model simulations were performed in MATLAB (version R2020b).

We simulated a population of 200 digital persons. For each simulated individual, the intrinsic period ($\tau$) was drawn from a known Gaussian distribution as reported in the literature^45^, while all other model parameters were kept fixed. We consider that an individual has an extremely short (or long) intrinsic circadian period if the drawn value lies more than one standard deviation $\sigma$ away from the population mean.

The Kronauer oscillator model requires time to stably entrain to a given light-dark cycle. As described in Ref.^48^, it has been observed that a period of 4 weeks is adequate to ensure consistent entrainment. To establish the baseline, we used 28 days of light data (30 minutes intervals) as the model input. We initiated the simulation on day 1 at the intended photoperiod/light pattern and run for 28 days for the entrainment before DST transitions. During the entrainment, the time of core-body temperature minimum gradually shifts towards an asymptote as illustrated in Fig. 7a. We consider the average time of reaching the minimum core body temperature during the fourth week as stable, which is utilized for determining the baseline circadian phase. We notice that when there is insufficient distinction between daytime ($L_1$) and evening ($L_2$) light, the model fails to entrain^27^. Results are presented only for combinations of daytime-evening illuminance settings and intrinsic circadian periods that the model entrains to 24-hour rhythms.

In order to model the DST transition, the initial 28 days are repeated but shifted by 1 hour (either back or ahead, depending on whether we model the spring or autumn DST transition). We consider that an individual is sufficiently adjusted to the new time zone if the deviation from the average CBT_min over the week prior to the transitions (baseline) is less than 10 minutes.

### Light scenarios

The various scenarios examined in our simulations are outlined in Table 1. For the first set of simulations (Fig. 2), evening light (19:00 to sleep) is fixed to 35 lx, and daytime light (wake to 19:00) is varied between 0 and 2,000 lx. The light profile is set to a fixed daytime level $L_1$ upon (self-selected) wake-up and changes to an evening light level $L_2$ starting at 19:00 and ending at the (self-selected) bedtime. We followed an approach similar to Ref.^27^, wherein the timing of light transitions depends on the model’s awakening and sleeping periods, thereby being ’self-selected’. The choice of 19:00 as the endpoint for daytime light was primarily based on the practical consideration that 19:00 marks a common transition to evening activities , many residents typically end their workday and return home around this time^49^.

The choice of daytime light levels was motivated by^50^ that reported typical horizontal (desk) illuminance levels in European offices ranging between 75 to 2,500 lx. In practical scenarios, the vertical illuminance reaching the cornea is expected to be considerably lower, typically ranging from 0.3 to 0.5 times the horizontal illuminance measured at desk level, depending on the directional properties of the light source(s)/fixture(s). While Smolders et al.^39^ reported an average illuminance of 200 lx measured vertically at the eye position during daytime hours, our findings in Fig. 9 suggest that the simulations with a daytime light intensity $L_1$ of 400 lx instead of 200 lx align better with the simulations based on the experimental light profile as obtained from our field study (see Fig. 8). Next to this, when assuming an melanopic daylight efficacy ratio of 0.63 for a typical mix of daylight and electric lighting within indoor exvironments, 400 lx vertically corresponds to a melanopic equivalent daylight illuminance of 250 lx as recommended by Brown et al.^51^ for health supportive daytime lighting.

The choice of evening light levels was influenced by the standard levels found in contemporary residential lighting settings (see Ref.^52^) with an average melanopic Effective Daylight Index (EDI) of 17.9 lx and a standard deviation (SD) of 13.6 lx. Assuming an M: P ratio (melanopic DER) of 0.52, this corresponds to a photopic illuminance of 35 lx^53,54^. Subsequently, we fixed daytime light (wake to 19:00) while varying evening light (19:00 to sleep) between 0 and 75 lx. The choice of evening light range was motivated by Ref.^52^ and a cross-country study among university students in China and Japan that reported even higher average levels of illuminance 2 hours before bedtime (74.8 lx ± 56.5 lx for the Japanese students and 59.9 lx ± 64.8 lx for the Chinese students)^55^.

**Table 1:** Scenarios and Illuminance Levels: different light settings employed in simulations, along with their corresponding illuminance levels and time intervals.

Scenario	Time interval	Illuminance [lx]	Type
A. Spring and Autumn DST transition with 35 lx evening light (Fig. 2)	Wake - 19:00	0 up to 2,000	$L_1$
	19:00 - Sleep	35	$L_2$
B. Spring DST transition varying evening & daytime light exposure (Fig. 3)	Wake - 19:00	200, 400	$L_1$
	19:00 - Sleep	0 up to 75	$L_2$
C. Morning walk (Fig. 5a)	Wake - Wake+60min	400	$L_1$
	Wake+60 min - Wake+90 min	10,000	$L_3$
	Wake+90 min - 19:00	400	$L_1$
	19:00 - Sleep	35	$L_2$
D. Evening walk (Fig. 5b)	Wake - 19:00	400	$L_1$
	19:00 - 19:30	2,000	$L_3$
	19:30 - Sleep	35	$L_2$

$L_1$ represents daytime light and $L_2$ represents evening light respectively.

## Data Availability

The light intensity data obtained from field study, which can be accessed by submitting a request to the corresponding author, specifying the purpose and scope of the research.
